# Stimuli-Responsive Biomass Cellulose Particles Being Able to Reversibly Self-Assemble at Fluid Interface

**DOI:** 10.3389/fchem.2020.00712

**Published:** 2020-10-06

**Authors:** Yue Zhu, Tingting Chen, Zhenggang Cui, Hong Dai, Li Cai

**Affiliations:** ^1^School of Chemistry and Chemical Engineering, Nantong University, Nantong, China; ^2^The Key Laboratory of Synthetic and Biological Colloids, Ministry of Education, School of Chemical and Material Engineering, Jiangnan University, Wuxi, China

**Keywords:** biomass cellulose particles, stimuli-responsive Pickering emulsions, *in situ* hydrophobization, reversibly self-assemble, amphiphiles

## Abstract

Stimuli-responsive surface-active microcrystalline cellulose (MCC) particles are obtained by interaction with conventional cationic surfactants such as cetyltrimethylammonium bromide (CTAB) in aqueous media, where MCC are *in situ* hydrophobized by adsorption of the cationic surfactant in water via electrostatic interaction and with the *in situ* hydrophobization removed by adding an equimolar amount of an anionic surfactant such as sodium dodecyl sulfate (SDS). The trigger is that the electrostatic interaction between the oppositely charged ionic surfactants is stronger than that between the cationic surfactant and the negative charges on particle surfaces, or the anionic surfactant prefers to form ion pairs with the cationic surfactants and thus making them desorbed from surface of MCC. Reversible O/W Pickering emulsions can then be obtained by using the MCC in combination with trace amount of a cationic surfactant and an anionic surfactant, and the anionic surfactant with a longer alkyl chain is more efficient for demulsification. With excellent biocompatibility, biodegradability, and renewability, as well as low toxicity, the biomass cellulose particles that can be made stimuli-responsive and able to reversibly self-assemble at fluid interface become ideal biocompatible particulate materials with extensive applications involving emulsions and foams.

**Graphical Abstract:**
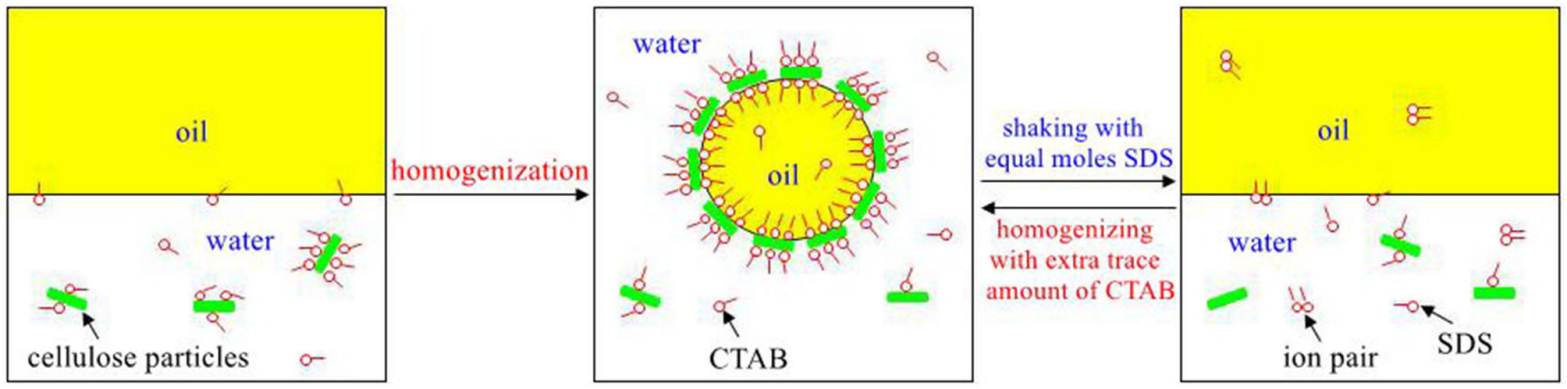


## Introduction

The emulsions stabilized by colloid particles are called Pickering emulsions. They are very stable because there is a dense particle film at the oil–water interface, providing a strong barrier to prevent coalescence of the droplets (Aveyard et al., 2003; Binks and Horozov, 2006). However, it is relatively difficult to demulsify when the emulsions are needed to be temporarily stable. Therefore, there has been much progress in preparing stimuli-responsible colloid particles, which can make the Pickering emulsions transit easily between stable and unstable. Various triggers have been developed such as pH (Binks et al., 2007; Fujii et al., 2011a,b; Liu et al., 2012, 2014; Morse et al., 2013; Tu and Lee, 2014), temperature (Binks et al., 2005; Saigal et al., 2010), CO_2_/N_2_ (Lin and Theato, 2013; Yang et al., 2013; Zhang et al., 2013; Liang et al., 2014; Liu et al., 2014), redox (Quesada et al., 2013), light irradiation (Anwar et al., 2013; Tan et al., 2014), and magnetic field (Lam et al., 2011; Blanco et al., 2013), as well as dual stimuli such as pH–temperature (Ngai et al., 2005; Yang et al., 2013; Yamagami et al., 2014), light–temperature (Fameau et al., 2013), and magnetic field intensity–temperature (Brugger and Richtering, 2007; Rahman et al., 2011). Nevertheless, the particles involved are mostly functional polymers, and their synthesis is relatively complicated. In recent years, commercially available inorganic nanoparticles with no surface activity in nature have been made surface-active by coating (Aveyard et al., 2003) or hydrophobized *in situ* (Cui et al., 2010, 2012). In addition, these inorganic particles such as silica nanoparticles can also be made stimuli-responsiveness through reversible hydrophobized *in situ* with the triggers including CO_2_/N_2_, pH, temperature, and ion pair formation (Jiang et al., 2013; Zhu et al., 2014, 2015a,b, 2017a; Liu et al., 2017).

However, the inorganic particles, lack of biocompatibility and biodegradability, are limited in the applications involving food, cosmetic, pharmaceutics, and so on. Therefore, recently, there is a great interest in biomass particles of biological origin for stabilizing emulsions and foams involved in food and drug delivery (Lam et al., 2014). The biomass particles include cellulose, starch, chitosan, and chitin, as well as aromatic macromolecules and polypeptides.

Cellulose is the most abundant biological polymer in the world, which is a linear polysaccharide with the units of β (1,4) glucopyranose. Cellulose particles have excellent biodegradability, renewability, and biocompatibility, as well as low toxicity. All these characteristics make them quite perfect materials in daily products including foods, cosmetics, and pharmaceutics (Lam et al., 2014). Many researchers have found that stable emulsions can be achieved by various sized cellulose including macroscopic fibers, microcrystalline cellulose (MCC), and nanofibrillated cellulose, in addition to cellulose nanocrystals (CNCs) as particulate emulsifiers in some certain conditions (Lam et al., 2014). It has also been found that there is significant difference in surface activity and film forming (Cherhal et al., 2016; Hu et al., 2016a; Varanasi et al., 2018; Alfassi et al., 2019; Costa et al., 2019) with cellulose of different origin, size, and surface property. The native cellulose is in general hydrophilic (not surface active), and the size and shape can be diverse (Lam et al., 2014); however, it has been reported that many hydrophobically modified cellulose particles have been made potentially applicable in foods, as well as drug delivery (Lam et al., 2014), thanks to great improvement of their surface activity by hydrophobic modification (Hu et al., 2015, 2016b; Ching et al., 2016; Duffus et al., 2016; Ojala et al., 2016; Tang et al., 2017; Zhu et al., 2017b; Bai et al., 2018, 2019; Aaen et al., 2019).

With hydrophilic surfaces, cellulose particles typically stabilize water-continuous emulsions, and oil-continuous emulsions have been achieved by Andresen and Stenius (Andresen and Stenius, 2007) by using silylation to improve the hydrophobicity and surface wettability of cellulose. But the surface activity endowed in this way is not reversible. In recent years, stimuli-responsive cellulose particles by suitable surface modification have also been reported (Zoppe et al., 2012; Tang et al., 2014, 2016, 2017). For example, Zoppe et al. (2012) made thermo-responsive CNCs by grafting poly(*N*-isopropylacrylamine) (PNIPAM) onto their surfaces; Tang et al. (2014) reported pH–temperature–responsive CNCs grafted by poly(dimetheylaminoethylmethacrylate), and then they further obtained another dual-responsive (pH and temperature) CNC nanoparticles based on grafting binary polymer brushes consisting of poly(oligoethylene glycol) methacrylate and poly(methacrylic acid) (Tang et al., 2016). Nevertheless, the synthesis of these particulate materials is complicated.

Herein we report that cellulose particles can be made surface-active by *in situ* hydrophobization in water via interaction with a cationic surfactant, and the surface activity of the particles can be switched off at room temperature to achieve reversible self-assemble at fluid interface. This is accomplished simply by adding an anionic surfactant of equimolar amount into the systems, forming ion pairs with the cationic surfactant and resulting in loss of surface activity of particles. The stimuli-responsiveness of the cellulose particles is characterized by stabilization and destabilization of emulsions, and both cationic and anionic surfactants with different chain length were examined for their efficiency in stabilization and demulsification.

## Experimental

### Materials

Microcrystalline cellulose (99%) with a primary particle diameter of 20 μm was purchased from Sigma. Cetyltrimethylammonium bromide (CTAB, 99%), dodecyltrimethylammonium bromide (DTAB, 98%), sodium dodecyl sulfate (SDS, 99%), sodium decyl sulfate (99%), and sodium octyl sulfate (99%) were all purchased from Sigma. Dodecane with a purity ≥99% was purchased from Aladdin and was columned two times through neutral alumina to remove possible polar impurities. Other chemicals were purchased from Sinopharm Chemical Reagent Co., which were all analytically pure. The ultrapure water used in all experiments with a resistance of 18.1 MΩ cm at 25°C was provided by Nantong University Analysis and Testing Center, China.

### Methods

#### Preparation Aqueous Dispersion of MCC Particles

Powdered MCC particles were weighed into a glass vessel with height of 6.5 cm and diameter of 2.5 cm, followed by adding pure water or surfactant solution. Then the particles were dispersed using an ultrasound probe (FS-250N; Shanghai ShengXi Co.) working at 50 W for 1 min.

#### Preparation and Characterization of Pickering Emulsions

The water phase (7 mL) containing MCC particles (dispersed in pure water or surfactant solution) was placed in a glass vessel, and then 1:1 by volume of dodecane (7 mL) was added. The two phases were emulsified using a A25 ultraturrax homogenizer (Shanghai OuHe Co.) operating at 7,000 revolutions/min (rpm) for 2 min.

The emulsion type was confirmed by drop test (Cui et al., 2010), and the photographs of the emulsions were taken 1 day and 7 days after preparation. To observe the microstructure of the emulsions, an emulsion drop was placed on a glass slide followed by diluted with water and then observed by a TL1530 microscope system (Shanghai DiLun Co.).

#### Demulsification/Restabilization Cycling of Emulsions

Emulsions stabilized by 0.3 wt% MCC dispersed in 0.01 mM cationic surfactant were destroyed by adding 0.07 mL concentrated (1 mM) anionic surfactant solution followed by gentle agitation with a stick and then leaving standing for 30 min. And the emulsions were restabilized again by addition of 0.07 mL concentrated (1 mM) cationic surfactant solution followed by homogenization at 7,000 rpm for 2 min.

#### Zeta Potential

Microcrystalline cellulose 0.3 wt% was dispersed in surfactant solutions or pure water of different pH adjusted by adding aqueous HCl or NaOH at 25°C. The dispersion was left on stand for 24 h to reach equilibrium; the zeta potentials (ζ) of the particles were measured using a Zetasizer Nano (Malvern) instrument at room temperature.

#### Interfacial Tension

The oil–water interfacial tension (IFT) was measured by drop shape method using Dropmeter A-100 drop shape analyzer (Ningbo Haishu Maishi Scientific Test Co., China), with the oil released from a U-shaped needle (outer diameter of 0.86 ± 0.005 mm) into water phase to form a reversed pendant drop at 25°C. Interfacial tension was calculated using the Young–Laplace method, and the result is an average of at least three measurements.

## Results and Discussion

### Dodecane-in-Water Pickering Emulsions Costabilized by Microcrystalline Cellulose and CTAB

**Figure 1:**
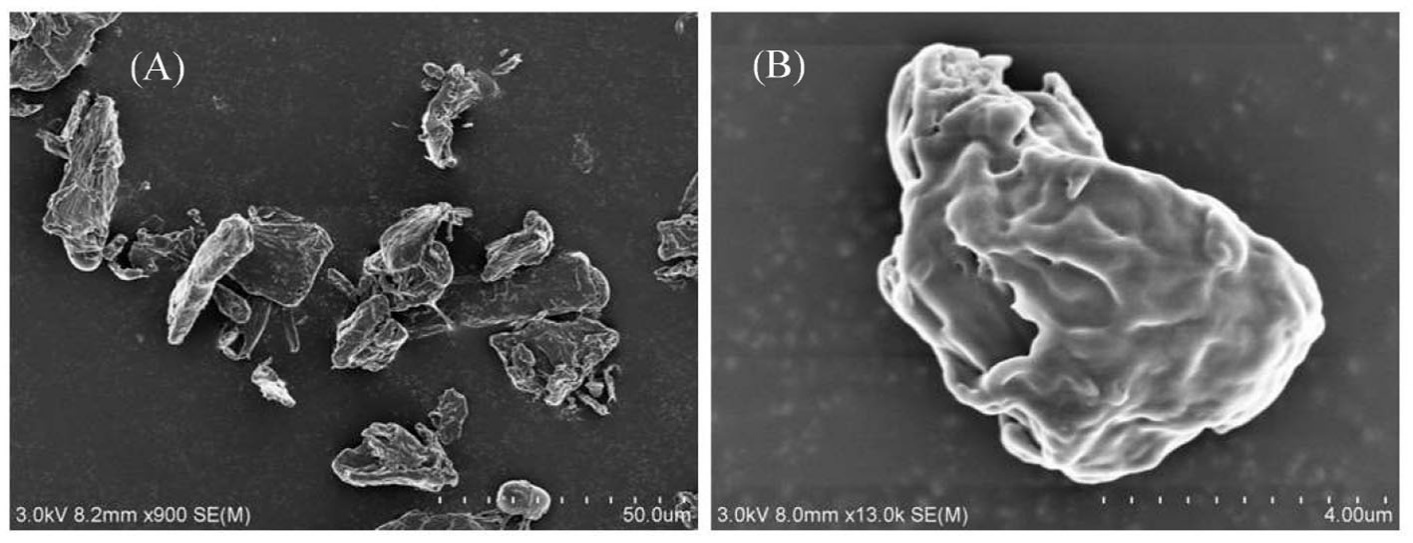
SEM of cellulose particles as powders **(A)** and was ultrasonically dispersed in water (0.3%) **(B)**.

**Figure 2:**
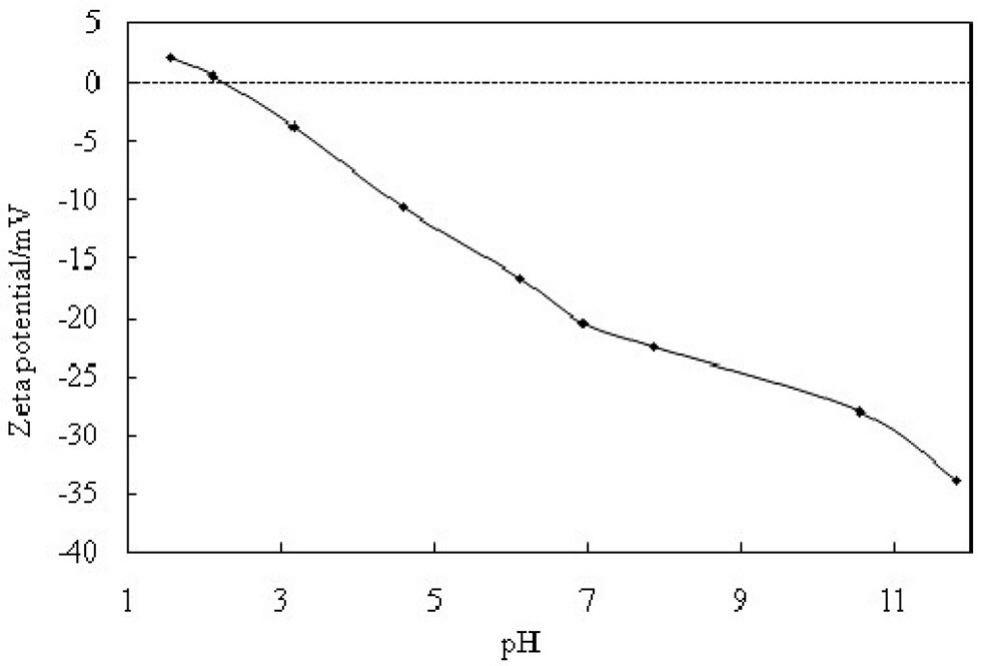
Zeta potentials of cellulose particles in water (0.3%) of different pH at 25°C.

**Figure 3:**
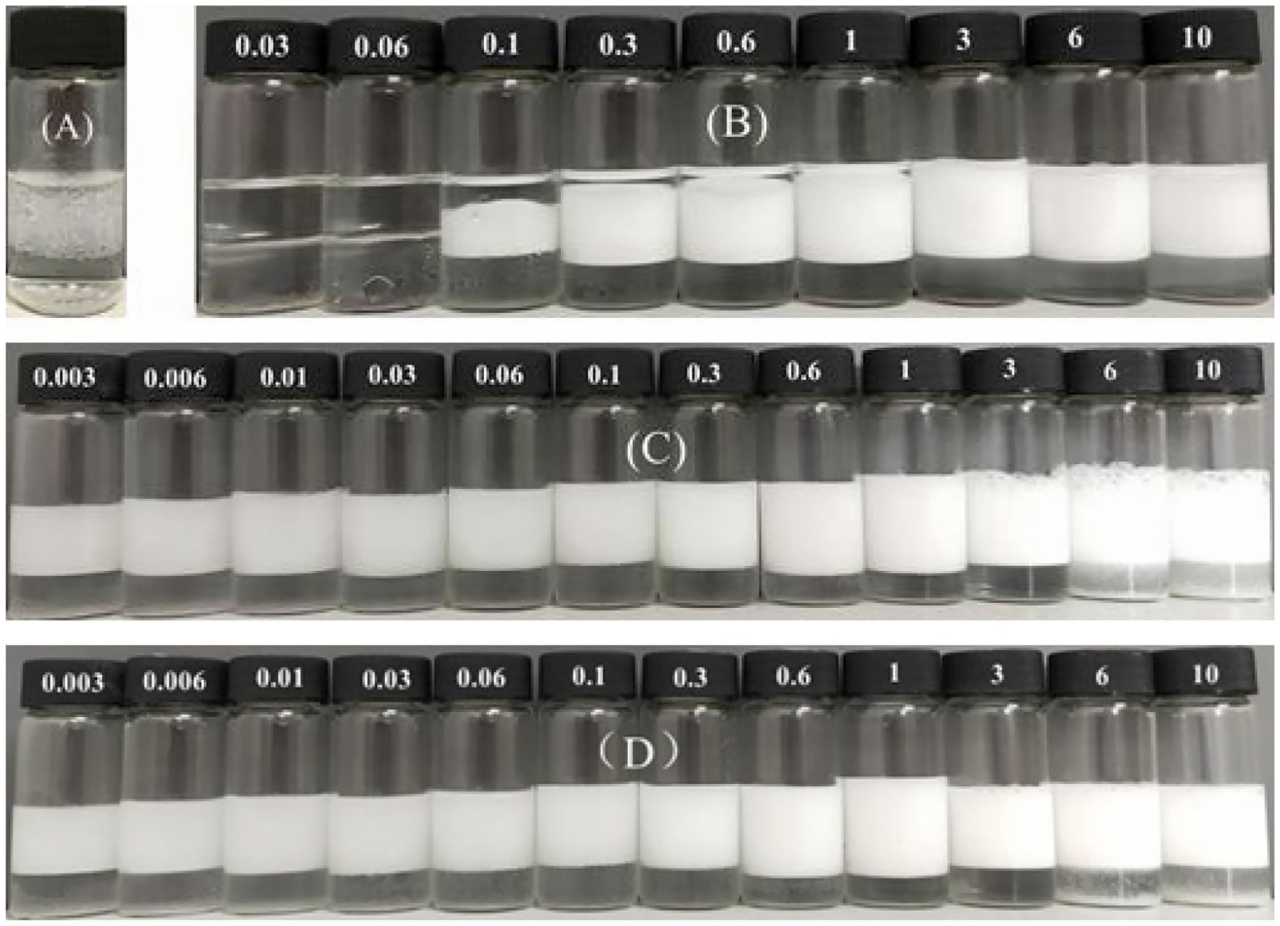
Dodecane-in-water emulsions stabilized by **(A)** 0.3 wt.% MCC particles solely, **(B)** CTAB alone at different concentration (mM), and 0.3 wt.% MCC particles plus CTAB at different concentration (mM), taken 24 h **(A–C)** and 7 days **(D)** after preparation.

**Figure 4:**
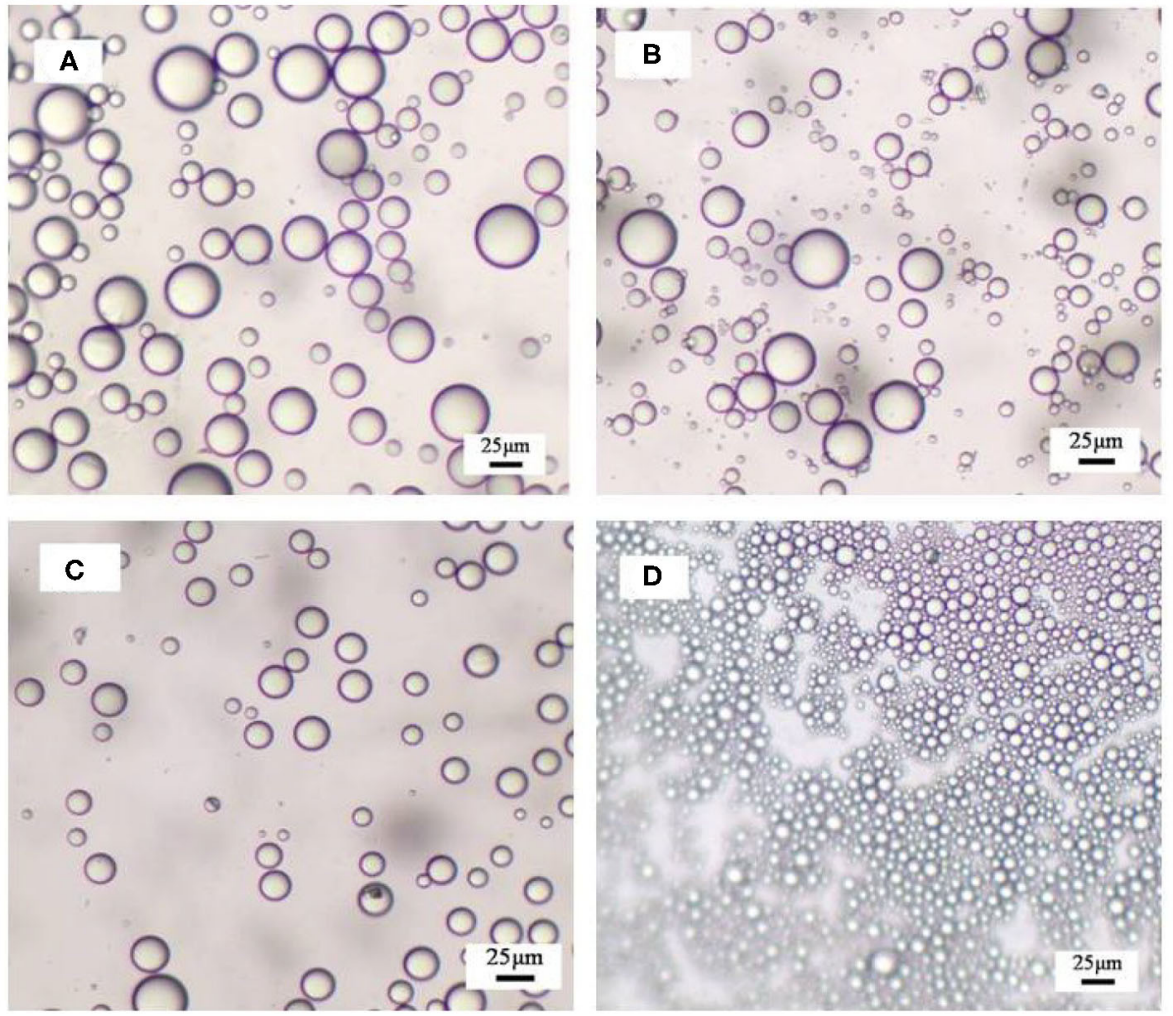
Optical micrographs of obtained emulsions stabilized by **(A–C)** 0.3 wt.% MCC together with CTAB of various concentrations and by **(D)** CTAB solely recorded 24 h after preparation. The concentrations of CTAB are 0.01, 0.06, 0.3, and 3 mM **(A–D)**.

The hydrophilic bare MCC particles are rod-like with a primary diameter 20 μm, as shown by the scanning electron microscope (SEM) image in Figure 1A. When ultrasonically dispersed in water, small particles with diameter of approximately 3–5 μm can be obtained as shown in Figure 1B. They have an isoelectric point of 2.2 (Figure 2) and is negatively charged in neutral water. Microcrystalline cellulose particles alone at 0.3 wt.% cannot stabilize a dodecane-in-water emulsion, with big drops observed in the vessel, as shown in Figure 3A. Similarly, CTAB alone below its critcal micelle concentration (cmc) of 0.9 mM (Zhu et al., 2015a) cannot stabilize a dodecane-in-water emulsion (Figure 3B). However, stable dodecane-in-water emulsions were obtained with 0.3 wt.% MCC particles plus CTAB, and almost no change appeared of the vessel after 24 h and 1 week as shown in Figures 3C,D. It was observed that the average droplet diameter decreases as CTAB concentration increases, as shown in Figure 4. It is noticed that all droplets are much bigger than those stabilized by CTAB solely at 3 mM (Figure 4D), indicating that these droplets are stabilized mainly by particles coated with surfactant, or the emulsions formed are Pickering emulsions.

### Destabilization/Restabilization Transition of the Pickering Emulsions

We have observed that if SDS, an anionic surfactant, was added (amount equal to that of CTAB) into an emulsion containing 0.3 wt.% MCC and CTAB of 0.01 mM, demulsification was observed with water phase and oil phase separated after gentle stirring. After an extra amount of CTAB (0.01 mM) was added into this system and the mixture was homogenized, stable Pickering emulsions can be formed again. This means that the Pickering emulsions stabilized by MCC and CTAB can be switched between stable and unstable, and MCC can be transferred between surface-active and surface-inactive.

**Figure 5:**
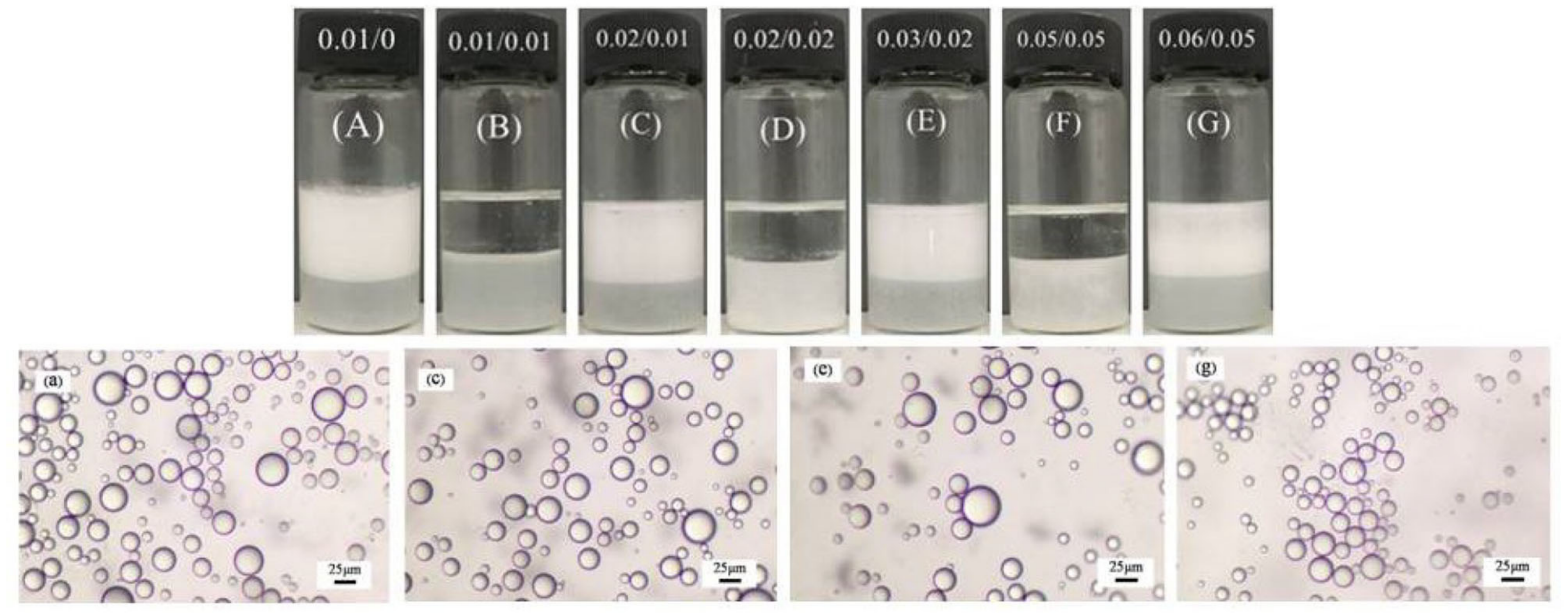
Photographs **(A–G)** of the dodecane-in-water Pickering emulsions stabilized by 0.3 wt.% MCC in combination with 0.01 mM CTAB undergoing unstable-stable cycles by adding 0.01 mM SDS and 0.01 mM CTAB alternately, and micrographs of the stable emulsions **(A–G)**.

For an emulsion comprising 0.3 wt.% MCC dispersed in 7 mL 0.01 mM CTAB solution and 7 mL dodecane (Figure 5A), once an equimolar amount of SDS (0.07 mL 1 mM SDS solution) was added (Figure 5B), almost complete demulsification was achieved after gentle agitation. Subsequently, when 0.01 mM free CTAB was added (adding extra 0.07 mL 1 mM CTAB solution), the stability of the emulsions was recovered by homogenization (Figure 5C). The emulsions are therefore stimuli-responsive by alternate addition of an equimolar amount of SDS and extra CTAB, respectively, and the system can be recycled for at least five times as shown in Figure 5. Based on the micrographs shown in Figure 5, the average droplet sizes were statistically measured to be 45, 45, 43, 41, and 40 μm, respectively, for the five cycles, little change compared with the initial emulsion, suggesting that the droplet sizes depend only on the concentration of free CTAB in the aqueous phase.

**Figure 6:**
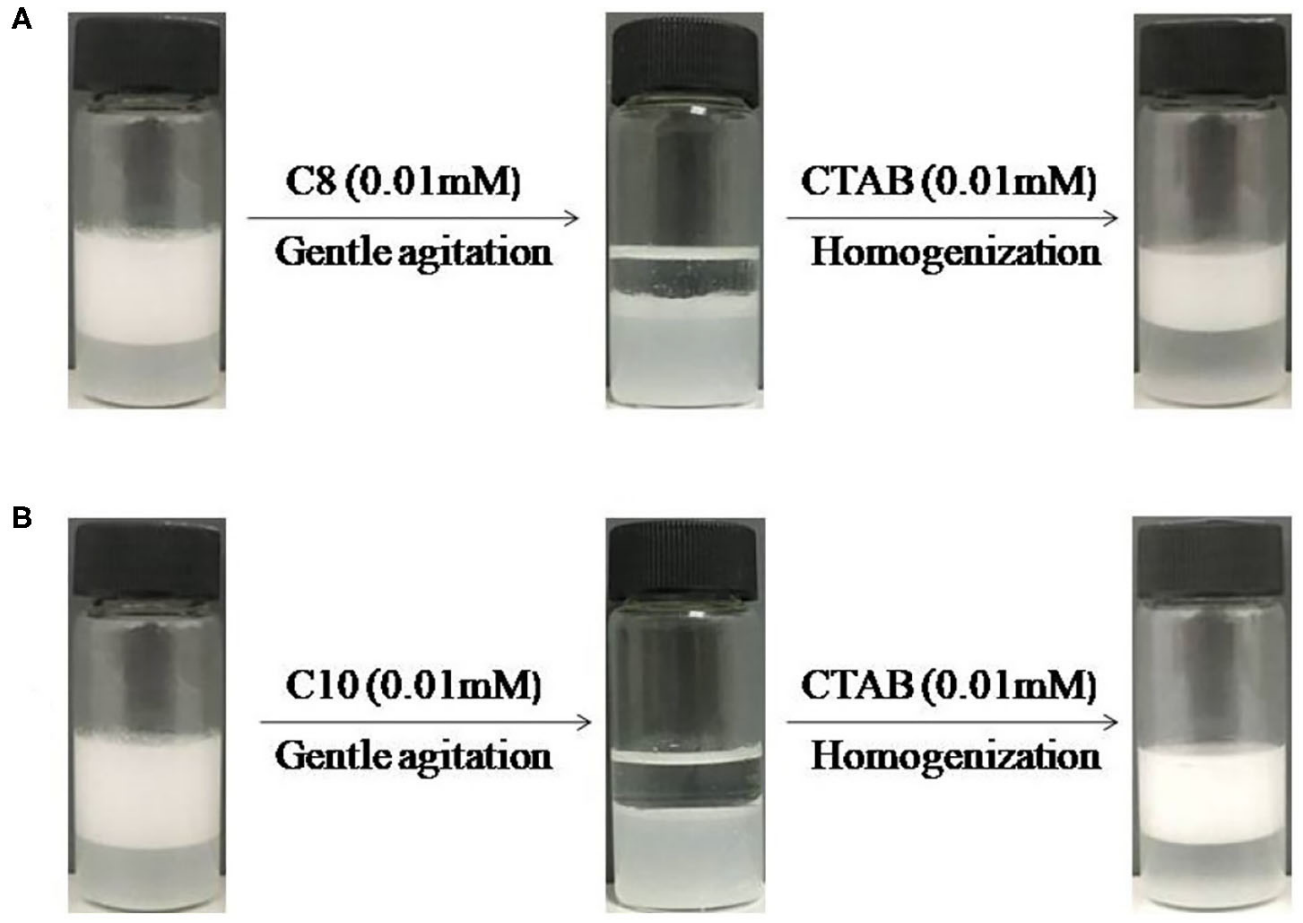
Switching dodecane-in-water emulsions containing 0.3 wt.% MCC with 0.01 mM CTAB between unstable by addition of **(A)** 0.01 M sodium octyl sulfate (C_8_) or **(B)** 0.01 M sodium decyl sulfate (C_10_) and restabilization by addition of extra 0.01 mM CTAB followed by homogenization.

**Figure 7:**
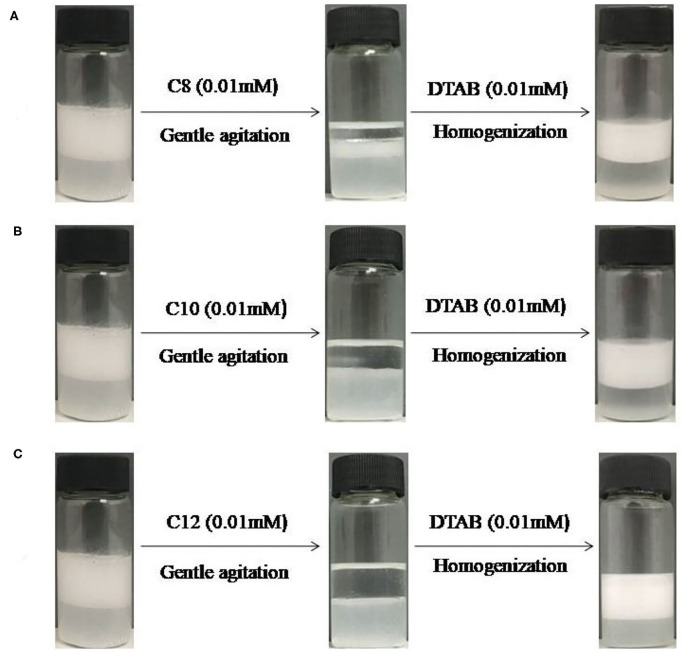
Switching between stable and unstable dodecane -in-water emulsions containing 0.3 wt% MCC with 0.01 mM DTAB followed by addition of (**A**) 0.01 M sodium octyl sulfate (C_8_) (**B**) 0.01 M sodium decyl sulfate (C_10_) (**C**) sodium dodecyl sulfate (C_12_, SDS) and subsequently 0.01 mM DTAB.

**Figure 8:**
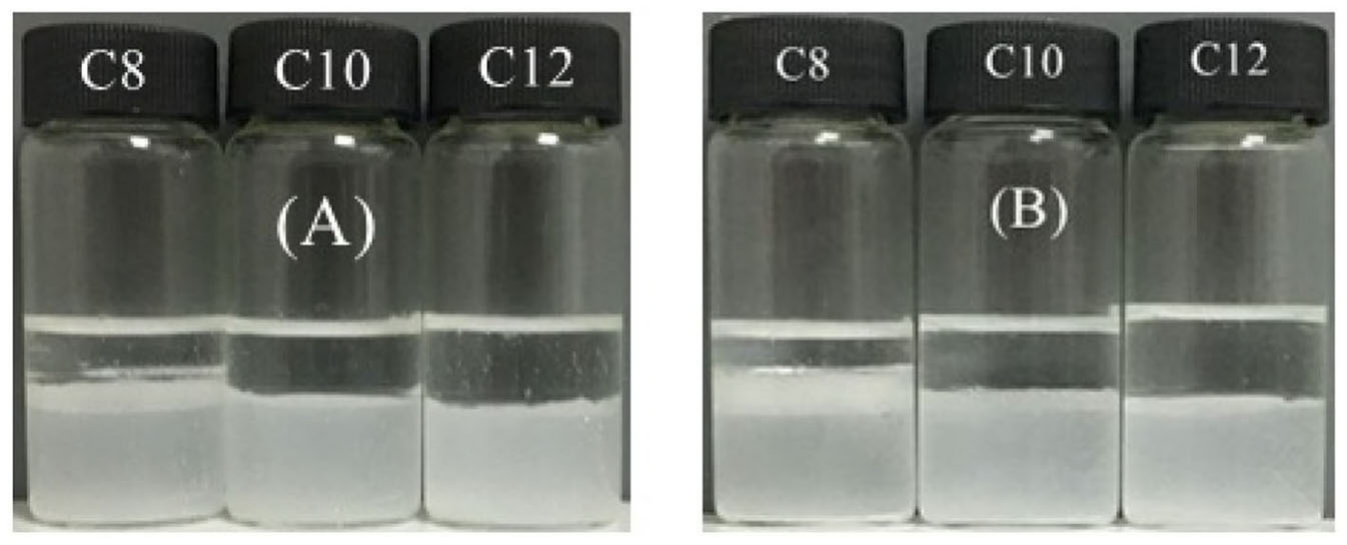
Demulsification of Pickering emulsions stabilized by 0.3 wt.% MCC plus 0.01 mM CTAB **(A)** or by 0.3 wt.% MCC plus 0.01 mM DTAB **(B)** by adding an equimolar amount of sodium alkyl sulfate of different chain lengths (given) followed by gentle agitation, taken 2 h after addition.

A question is whether other cationic surfactants and anionic surfactants are effective for *in situ* hydrophobization of the cellulose particles and demulsification. To find answers, DTAB as cationic surfactant and a series of sodium alkyl sulfates (C_8_-C_12_) as anionic surfactants were examined. Figure 6 shows that in case of CTAB as cationic surfactant both C_8_ and C_10_ sodium alkyl sulfates are also effective at 0.01 mM for demulsification. And once CTAB was replaced by DTAB (C_12_) stimuli-responsible emulsions were obtained using C_8_ to C_12_ sodium alkyl sulfates as demulsifiers, as shown in Figure 7. However, the alkyl length did affect demulsification efficiency, as shown in Figure 8, where the oil phase is clearer after demulsification with increasing alkyl length of the anionic surfactants, and demulsification is not complete when using sodium octyl sulfate (C_8_), because the tendency of forming ionic pair increases with increasing total alkyl length. In fact, we have previously reported that for negatively charged silica nanoparticles the total alkyl length of cationic and anionic surfactants should be larger than C_22_ (Zhu et al., 2015a); this seems to be also true for the cellulose particles.

### Postulated Mechanism of MCC Reversibly Self-Assembling at Fluid Interface

**Figure 9:**
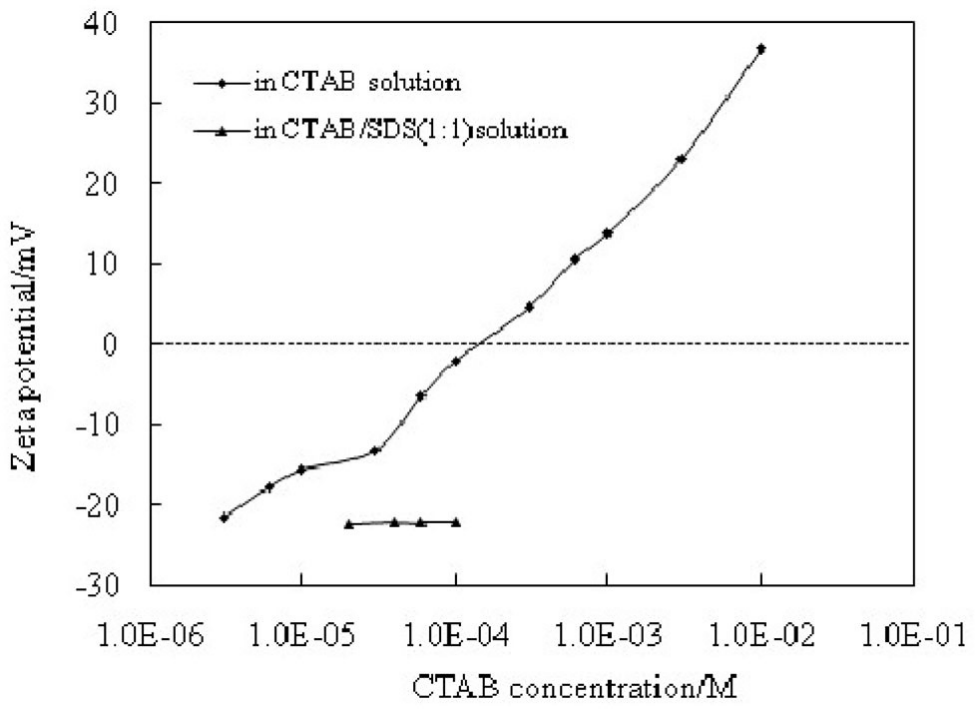
Zeta potentials of 0.3 wt.% MCC particles that were dispersed in aqueous solutions of CTAB and in solutions containing equimolar amount of CTAB + SDS.

The stabilization and destabilization of emulsions indicate that MCC particles can reversibly self-assemble at fluid interface. The MCC particles are too hydrophilic, which has negatively charged when pH is beyond 2.2, as indicated in Figure 2. They are little surface-active with a zeta potential of −22.5 mV in the pure water (pH of dispersion 7.86). When MCC particles are dispersed in CTAB solution, the zeta potential is increased with increasing the concentration of CTAB, from negative to positive (Figure 9). It is proved that the cationic surfactant, CTAB, adsorbs to the negatively charged surfaces of MCC particles via electrostatic interaction and thus *in situ* hydrophobizes the surfaces. The particles then become surface-active to adsorb at the water-oil interface, stabilizing the Pickering emulsion, whereas, on the addition of SDS, an anionic surfactant, CTAB, prefers to form ion pairs with SDS (Kume et al., 2008; Tah et al., 2011), which makes CTAB desorb from MCC particles, resulting in demulsification as MCC particles return to the aqueous phase and become surface-inactive again. The mechanism is that there is much stronger electronic interaction between the anionic and cationic surfactants than that between particles and cationic surfactants with opposite charge. Here we provide the evidence of zeta potential, SEM, and the IFT to support the theory.

**Figure 10:**
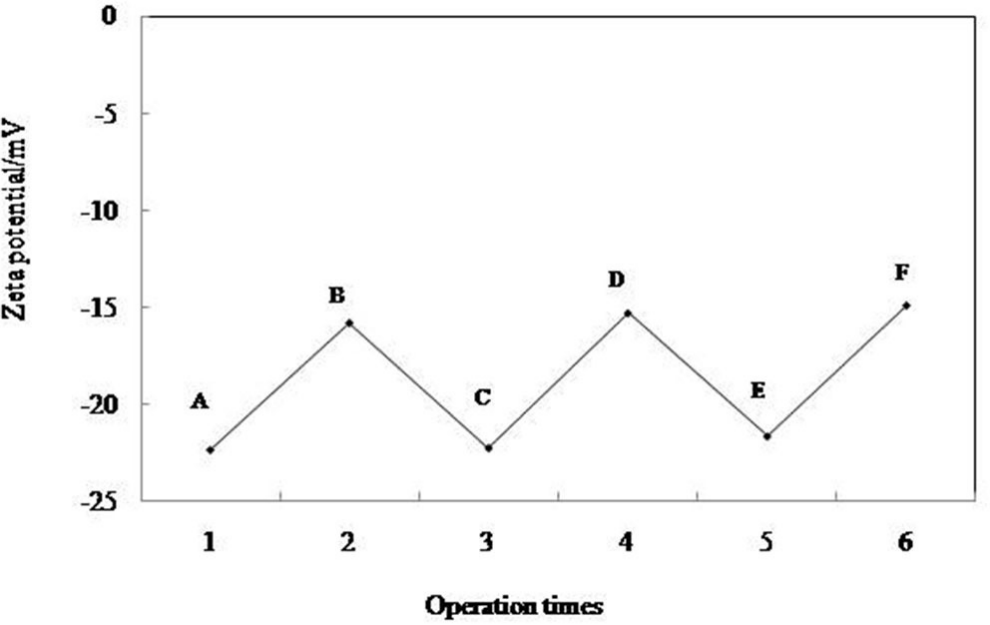
Zeta potentials of 0.3 wt. % MCC particles dispersed in pure water (A), in 0.01 mM CTAB solution (B), after cycles of addition equimolar amount of SDS (C,E), and extra amount of 0.01 mM CTAB (D,F).

The change of zeta potential of 0.3 wt.% MCC during emulsification and demulsification cycling is shown in Figure 10. When 0.3 wt.% MCC is dispersed in the pure water, the zeta potential is −22.5 mV (Figure 10A), and then it increases to −15.8 mV (Figure 10B) in 0.01 mM CTAB solution. After addition of equimolar amount of SDS and an extra CTAB of 0.01 mM, respectively, the zeta potential decreases to −22.3 mV (Figure 10C) and increases to −15.3 mV (Figure 10D) in the first cycle and becomes −21.7/−14.9 mV (Figures 10E,F) in the second cycle, which indicates that the adsorption/desorption of the surfactant from the particle–water interface following adding CTAB and SDS is reversible. It is also indicated by the photographs and micrographs shown in Figure 5. All these prove that cellulose particles can reversibly self-assemble at fluid interface.

Besides, the zeta potential of 0.3 wt.% MCC in the solution containing equimolar amount of SDS and CTAB at different concentration is almost not changed (−22.3 ± 0.1 mV) and is the same as in the pure water. It is believed that ion pairs consisting of anionic and cationic surfactants cannot absorb to the particles, as shown in Figure 9. No stable emulsions were observed using this dispersion because particles are surface-inactive.

**Figure 11:**
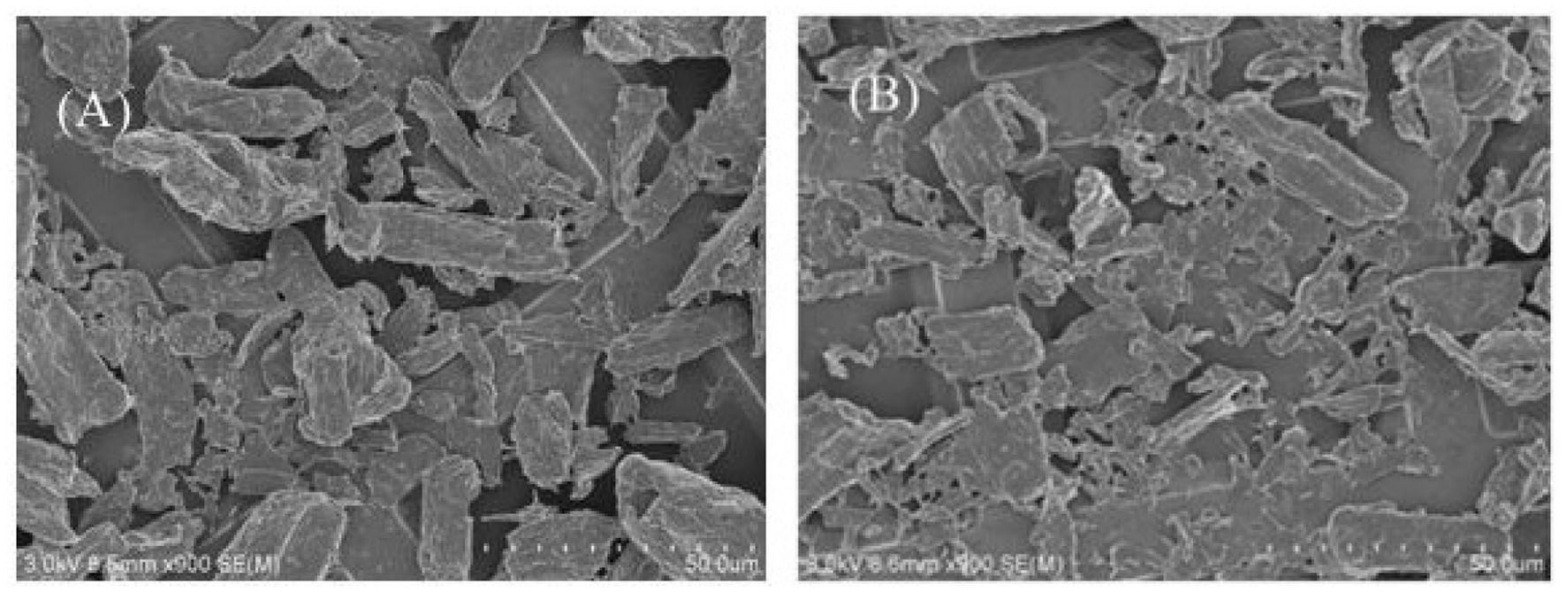
SEM of 0.3 wt.% MCC dispersed in 0.01 mM CTAB solution **(A)** and in CTAB + SDS equimolar mixture at 0.01 mM **(B)**.

We have measured the dodecane/water IFT with either particles or surfactants or both in water, as shown in Table 1. The dodecane/pure water IFT is in good agreement with literature value (52.1 mN/m). Although the SEM of 0.3 wt.% MCC dispersed in 0.01 mM CTAB solution and in CTAB + SDS equimolar mixture at 0.01 mM shows no significant difference (Figure 11); the IFT (41.8 mN/m) between dodecane and dispersion of 0.3% particle in 0.01 mM CTAB solution is higher than that (35.8 mN/m) between dodecane and 0.01 mM CTAB solution, indicating adsorption of CTAB on particle surface, which reduced CTAB concentration in the dispersion. Actually flocculation was observed when 0.3 wt.% cellulose particles were dispersed in CTAB aqueous solution at CTAB concentration beyond 0.3 mM (not shown). When equal moles of SDS were added (0.3% cellulose particles dispersed in 0.01 mM CTAB + 0.01 mM SDS solution), the IFT (39.0 mN/m) is between the previous two systems. It is believed that the formation of ionic pairs reduces significantly the concentration of free CTAB and SDS, but the ionic pairs are also highly surface-active, which can strongly adsorb at oil–water interface to reduce the IFT.

**Table 1:** Interfacial tension between dodecane and aqueous phase with or without particles and surfactants at 25°C.

**No**.	**Composition of aqueous phase**	**γ/mN/m**
1	Pure water	52.1 ± 0.1
2	0.3% Cellulose	48.7 ± 0.1
3	0.01 mM CTAB	35.8 ± 0.2
4	0.3% Cellulose + 0.01 mM CTAB	41.8 ± 0.2
5	0.3% Cellulose + 0.01 mM CTAB+0.01 mM SDS	39.0 ± 0.2

## Conclusions

A simple protocol has been demonstrated to prepare the stimuli-responsive surface-active MCC particles, which are able to reversibly self-assemble at fluid interface via reversible *in situ* hydrophobization to stabilize stimuli-responsive Pickering emulsions. The stable Pickering emulsion is obtained by using negatively charged MCC particles *in situ* hydrophobized with a cationic surfactant in low concentration, whereas demulsification occurs by adding an anionic surfactant of equal moles. The restabilization of the Pickering emulsion is achieved again on addition of extra cationic surfactant which reestablishes the hydrophobization. The stimuli-responsiveness of the emulsions is due to the trigger that the electrostatic interaction between the oppositely charged ionic surfactants is stronger than that between the cationic surfactant and the particle surfaces. The added anionic surfactant prefers to form ion pairs, making cationic surfactant desorb from particle surfaces and particles surface-inactive returning to the water aqueous. This access avoids complicated synthesis of functional switchable particles as well as relative rigorous switching conditions. With excellent biocompatibility, biodegradability, and renewability, as well as low toxicity, the biomass cellulose particles, which are made stimuli-responsive and can then reversibly self-assembling at fluid interface, become ideal biocompatible particulate materials with more potential applications in many fields.

## Data Availability Statement

The raw data supporting the conclusions of this article will be made available by the authors, without undue reservation.

## Author Contributions

YZ wrote the manuscript. HD made the additional experiments according to the comments of reviewer. LC tested particles and Pickering emulsion. TC made the stimuli-responsiveness cellulose particles. ZC revised the article. All authors contributed to the article and approved the submitted version.

## Conflict of Interest

The authors declare that the research was conducted in the absence of any commercial or financial relationships that could be construed as a potential conflict of interest.
